# Adverse Events among HIV/MDR-TB Co-Infected Patients Receiving Antiretroviral and Second Line Anti-TB Treatment in Mumbai, India

**DOI:** 10.1371/journal.pone.0040781

**Published:** 2012-07-11

**Authors:** Petros Isaakidis, Bhanumati Varghese, Homa Mansoor, Helen S. Cox, Joanna Ladomirska, Peter Saranchuk, Esdras Da Silva, Samsuddin Khan, Roma Paryani, Zarir Udwadia, Giovanni Battista Migliori, Giovanni Sotgiu, Tony Reid

**Affiliations:** 1 Médecins Sans Frontières, Mumbai, India; 2 Médecins Sans Frontières, South African Medical Unit (SAMU), Cape Town, South Africa; 3 University of Cape Town, Cape Town, South Africa; 4 Parmanand Deepchand Hinduja National Hospital and Medical Research Centre (Hinduja), Mumbai, India; 5 World Health Organization Collaborating Centre for Tuberculosis and Lung Diseases, Fondazione S. Maugeri, Care and Research Institute, Tradate, Italy; 6 Epidemiology and Medical Statistics Unit, Department of Biomedical Sciences, University of Sassari, Sassari, Italy; 7 Médecins Sans Frontières, Operational Research Unit, Brussels, Belgium; Institute of Infectious Diseases and Molecular Medicine, South Africa

## Abstract

**Background:**

Significant adverse events (AE) have been reported in patients receiving medications for multidrug- and extensively-drug-resistant tuberculosis (MDR-TB & XDR-TB). However, there is little prospective data on AE in MDR- or XDR-TB/HIV co-infected patients on antituberculosis and antiretroviral therapy (ART) in programmatic settings.

**Methods:**

Médecins Sans Frontières (MSF) is supporting a community-based treatment program for drug-resistant tuberculosis in HIV-infected patients in a slum setting in Mumbai, India since 2007. Patients are being treated for both diseases and the management of AE is done on an outpatient basis whenever possible. Prospective data were analysed to determine the occurrence and nature of AE.

**Results:**

Between May 2007 and September 2011, 67 HIV/MDR-TB co-infected patients were being treated with anti-TB treatment and ART; 43.3% were female, median age was 35.5 years (Interquartile Range: 30.5–42) and the median duration of anti-TB treatment was 10 months (range 0.5–30). Overall, AE were common in this cohort: 71%, 63% and 40% of patients experienced one or more mild, moderate or severe AE, respectively. However, they were rarely life-threatening or debilitating. AE occurring most frequently included gastrointestinal symptoms (45% of patients), peripheral neuropathy (38%), hypothyroidism (32%), psychiatric symptoms (29%) and hypokalaemia (23%). Eleven patients were hospitalized for AE and one or more suspect drugs had to be permanently discontinued in 27 (40%). No AE led to indefinite suspension of an entire MDR-TB or ART regimen.

**Conclusions:**

AE occurred frequently in this Mumbai HIV/MDR-TB cohort but not more frequently than in non-HIV patients on similar anti-TB treatment. Most AE can be successfully managed on an outpatient basis through a community-based treatment program, even in a resource-limited setting. Concerns about severe AE in the management of co-infected patients are justified, however, they should not cause delays in the urgently needed rapid scale-up of antiretroviral therapy and second-line anti-TB treatment.

## Introduction

Even though treatment for multidrug-resistant and extensively-drug-resistant tuberculosis (MDR-TB & XDR-TB) and antiretroviral therapy (ART) have been shown to improve patient outcomes, treatment of MDR-TB in HIV-infected patients remains a significant challenge [1]–[4]. Such patients are required to take large numbers of pills each day, receive intramuscular injections for extended periods of time, and are subject to the potential additive side effects and drug interactions between antiretroviral agents and second-line anti-tuberculosis drugs [5], [6].

To date, very few studies have reported on ambulatory MDR-TB treatment adverse events (AE) [3], [4], [7]–[12]. In addition, there is a more serious deficiency in reports describing AE of MDR-TB treatment in HIV-infected patients, especially in programmatic settings in resource-constrained countries.

Médecins Sans Frontières (MSF) has been treating MDR-TB among HIV-infected individuals in Mumbai since May 2007. MDR-TB treatment became available in the public sector in Mumbai only in late 2010.

This report aims to describe the occurrence of AE in HIV-infected patients on antiretroviral and MDR-TB treatment. We also aim to establish the extent of resolution of the AE after management, and appropriate frequency of monitoring to allow early detection of AE during the course of treatment.

## Methods

### Study design

This was a prospective, observational cohort study using data routinely collected at each consultation and entered into patient files and electronic databases.

### Setting and study population

MSF has been operating an HIV clinic in Mumbai, India since 2006. An MDR-TB component was added to the HIV treatment program in May 2007. All HIV-infected patients with confirmed MDR-TB or suspected to have MDR-TB based on clinical findings and TB treatment history, and who were started on both ART and MDR-TB treatment between May 2007 and September 2011, were included in this study. The patients were included from initiation of their MDR-TB treatment to determination of their MDR-TB treatment outcome, as different types of AE are more or less likely to occur during different periods of the treatment.

### Treatment protocol and follow-up

All patients received individualized therapy through an ambulatory, community-based program that we have described elsewhere [4]. In summary, an individualized treatment regimen was designed for each patient, based on the first and second line drug susceptibility test (DST) results and on a patient's treatment history. A standardized treatment regimen was used for empiric treatment in those patients who required immediate treatment initiation due to the severity of their disease, or in those for whom TB culture was negative, but MDR-TB was strongly suspected based treatment history and/or history of contact with confirmed MDR-TB patients. The standardized regimen included six drugs: pyrazinamide, capreomycin, moxifloxacin, ethionamide, cycloserine and *p*-aminosalicylic acid (PAS). The standardized regimen was modified once the DST results became available, or was continued as such for culture-negative patients. Patients were treated for 18 months in the initial stages of the program, based on the WHO guidelines at that time [5]. Currently, patients are treated for at least 20 months, based on WHO guidelines updated in 2011 [13].

Patients were evaluated by a multidisciplinary team of trained physicians, nurses, social workers and a psychologist at baseline, and the same team followed the patients throughout their treatment. The attending physician examined patients clinically every two weeks during the first month of treatment and once a month thereafter. At the community level, a network of public and private health structures and non-governmental organizations (NGOs) acted as providers of directly observed therapy (DOT). The DOT providers were trained to support the patients and to report adverse events.

At each clinic visit, the patient was assessed clinically for treatment response and AE, as per the clinic protocol. Table 1 shows the AE definitions and grading used in the clinic, as well as the monitoring tools, the frequency of monitoring and the basic principles of their management. The severity of AE was defined by laboratory criteria (whenever quantifiable) or based on effect on patient tolerance and adherence. AE were aggressively managed in the following order, advancing to the next step if no relief was obtained from the preceding intervention: counseling and symptomatic treatment, splitting of the total dose of the suspected offending drug from once daily to twice daily, reduction of the total dose of the medication by one weight class, and drug substitution of the suspected offending drug. For very severe or life-threatening events, this order was not followed.

**Table 1:** Definitions, grading, monitoring and management of adverse events in MSF HIV/MDR-TB program, Mumbai, India.

Adverse event	Definition/Grading	Likely drug	Test/Frequency	Management
Gastrointestinal symptoms	Any documentation of nausea and/or vomiting and/or diarrhoea by physician **Mild:** self limited, symptomatic management **Moderate:** requires TB-drug dose modification**Severe:** requires substitution of suspect drug	PAS, P, E, Ethio, AZT, LPV	Clinical/Monthly	Antiemetic (metoclopramide; ondansetron if severe). Anti-gastritis (mucaine gel or omeprazole). Anti-diarrheal medication. Splitting of dose of ethionamide if above measures do not help. Giving PAS with yogurt, coconut water or fruit juice. Lowering dose of PAS to 7–8 gm BD if above measures do not help.
Peripheral neuropathy	Symptoms and findings consistent with neuropathy, as diagnosed by physician or by electromyography **Mild:** responds to Pyridoxine only **Moderate:** Amitryptyline needs to be added **Severe:** Gabapentene needs to added	INH (high dose), Cs, E Ethio, D4T	Clinical/Monthly	Pyridoxine 50mg/250mg of Ethionamide and/or Cycloserine added for all patients at treatment initiation. Amitryptyline, Gabapentene for ascending severity of symptoms
Hypothyroidism	Serum thyroid stimulating hormone (TSH) greater than 10 mIU/L **Mild:** asymptomatic, TSH >10mIU/L **Moderate:** Symptomatic hypothyroidism **Severe:** Myxedema, coma	Ethio, PAS	T3, T4, TSH/Baseline, 3, 6, 10, 12, 14, 18, 21, 24 months	Levothyroxine at TSH >10 mIU/L OR at low values of thyroid hormones
Psychiatric disturbances	Presence of one or more of the following: depression, anxiety, and/or psychotic symptoms as defined by DSM IV criteria and/or as evaluated by a psychiatrist **Mild:** symptomatic management **Moderate:** need for TB-drug dose modification. **Severe:** requires substitution of culprit drug	Cs, EFV(FQ, Ethio)	Baseline psychiatric assessment/ Monthly clinical (by psychologist)	Pyridoxine 50mg/ 250mg of Cycloserine added for all patients at treatment initiation. Individualized management by psychiatrist and psychologist.
Hypokalemia	At least one serum potassium value of <3.0 mmol/l **Mild:** serum potassium >2.4 mmol/l **Moderate:** serum potassium 2.0–2.4 mmol/l **Severe:** serum potassium <2.0 mmol/l	Cm, K, S	Serum potassium/ Baseline, monthly for full duration of injectable use	Food supplements at low values but >3 mmol/l, K+ Mg supplements if <3 mmol/l, hospitalization of symptomatic patients
Renal impairment	At least one value CrCl <30 ml/min **Mild:** 60> CrCl >50 **Moderate:** CrCl = 30–50 **Severe:** CrCl <30	K, Cm, S TDF	Serum creatinine/ Baseline, monthly for whole duration of IP	Adjustment of drug doses as per WHO guideline at CrCl <30 ml/min
Auditory toxicity	Hearing loss self-reported and confirmed by audiometry **Mild:** symptomatic (self-reported) **Moderate:** need for dose modification **Severe:** requires substitution of culprit drug	K, Cm	Audiometry/Baseline audiometrymonthly history for full duration IP	Referral to otorhinolaryngologist. Change to less ototoxic drug preferred over lowering of dose
Seizures	**No grading.** Considered severe and requires substitution of culprit drug if not controlled by anticonvulsant	Cs, INH (high dose), FQ	N/A	Anticonvulsants
Arthralgia	Self-reported pain **Mild:** self-limited **Moderate:** symptomatic management **Severe:** requires substitution of culprit drug	Z, FQ	N/A	Antinflammatory agents
Vestibular toxicity	Symptoms and findings consistent with vestibular toxicity, e.g. vertigo and/or loss of balance **Mild:** self- limited **Moderate:** symptomatic management **Severe:** requires substitution of culprit drug	K, Cm	Clinical/Monthly for full duration of injectable use	Referral to otorhinolaryngologist. Anti-vertigo drugs and/or change to less ototoxic drug preferred over lowering of dose
Tendinitis	**No grading**	FQ		Requires substitution of culprit drug
Hepatitis	Elevation of ALT to more than twice the upper limit of the normal values/clinical jaundice irrespective of ALT value **Mild:** ALT 50– 100 U/L **Moderate:** 100 –200 U/L **Severe:** >200 U/L	All, most likely PZA, NVP, (Ethio, PAS, FQ, EFV, ritonavir)	ALT/Baseline, 2-wkly in 1^st^ month, monthly in IP, 3-mthly in CP	Individualized

IP: intensive phase, CP: continuation phase, D4T  =  stavudine; Cs  =  cycloserine; INH = isoniazide, E  =  ethambutol; Ethio  =  ethionamide; AZT  =  azidothymidine; TDF  =  tenofovir; EFV  =  efavirenz; FQs  =  fluoroquinolones; LPV+lopinavir; NVP  =  nevirapine; P  =  pyrazinamide; PAS  =  para-aminosalicylic acid; ALT  =  alanine aminotransferase; ARV  =  antiretroviral; S  =  streptomycin; K  =  kanamycin; Cm  =  capreomycin.

Patients, unless already on ART, were started on antiretroviral drugs as soon as they were tolerating second-line TB drugs, irrespective of CD4 cell count. Two nucleoside/tide reverse transcriptase inhibitors (NRTIs) and one non-nucleoside reverse transcriptase inhibitor (NNRTI) were used for patients being prescribed first line ART, while patients in need of second line ART received a protease inhibitor-based regimen along with NRTIs [14].

### Data collection and analysis

Demographic and clinical information were systematically recorded in standardized clinical files designed specifically for the program. Information on all patients was prospectively collected and entered into an electronic database. Information on HIV and antiretroviral therapy was collected in the same patient file but entered in a separate database. Each patient had a unique identification code that was used in both databases. Trained personnel regularly extracted clinical, treatment, and laboratory data from individual patient records and entered them into both databases. A full time data manager routinely checked data entry for accuracy and completeness.

Data from all patients started on MDR-TB treatment between May 2007 and September 2011 were included in the analyses. Patient characteristics at admission into the MDR-TB program and the occurrence of AE up to the end of the study period were summarized using descriptive statistics. We used chi-square and Fisher's exact test to assess differences between categorical variables. Univariate and multivariate models were fitted to assess risk factors associated with the occurrence of adverse events. Factors associated with AE in the univariate analysis at p<0.20 were selected for inclusion in the multivariate logistic regression analysis. We also tried to assess whether the occurrence of severe AE was associated with unfavorable treatment outcomes (defined as death, default, or treatment failure) [15]. A p-value of less than 0.05 was considered to indicate statistical significance. The computer software SPSS (version 16.0, Chicago, IL) was used for analysis.

### Ethics

The study satisfied the criteria for reports using routinely-collected programmatic data, set by the Médecins Sans Frontières independent Ethics Review Board, Geneva, Switzerland. As this was a study of routinely collected monitoring data, informed consent from the patients was not obtained. The named ethics committee specifically approved the study and waived the need for consent.

## Results

### Patient characteristics

Between May 2007 and September 2011, 81 HIV-infected patients were diagnosed with MDR-TB (67 bacteriologically confirmed and 14 unconfirmed) and registered in the MSF clinic. Of these, 67 HIV/MDR-TB co-infected patients (53 bacteriologically confirmed and 14 unconfirmed) who had received at least two weeks of anti-MDR-TB therapy were included in subsequent analyses. Twenty-nine (43.3%) of them were female and two (3.0%) were transgender individuals. The median age of these patients was 35.5 years (Interquartile Range [IQR] 30.5–42) and the median duration of MDR-TB therapy at the time of analysis was 10 (range 0.5–30) months.

### Tuberculosis and HIV clinical characteristics and treatment history

Overall, 59 patients (88.1%) had pulmonary TB, twelve of whom were diagnosed with both pulmonary and extra-pulmonary TB. Three patients were diagnosed with extensively drug-resistant TB (XDR-TB). All but five patients (92.5%) had received previous TB treatment, half in the public sector and half in the private or both sectors. Half of the patients had a history of previous exposure to second-line TB drugs, most commonly fluoroquinolones. Table 2 shows the drugs prescribed, the maximum doses and the number of patients per prescribed drug. The median duration of injectable drug use was six months (range 0.5–24).

**Table 2:** Treatment regimens of HIV-infected MDR-TB patients (n = 67).

Medication/Maximum dosage	Patients on each drug *n (%)
**TB-drugs**	
Rifampicin (RMP) 600 mg/day	4 (6)
Isoniazid (INH) 20 mg/kg/day of body weight	19 (28)
Pyrazinamide (PZA) 40 mg/kg/day	43 (64)
Ethambutol (EMB) 25 mg/kg/day	15 (22)
Streptomycin (S) 1 g/day	3 (4)
Kanamycin (K) 1 g/day	38 (57)
Capreomycin (Cm) 1 g/day	38 (57)
Levofloxacin (LFX) 750 mg/day	25 (37)
Moxifloxacin (MFX) ) 400 mg/day	51 (76)
Ethionamide (ETH) 1000 mg/day	52 (78)
Cycloserine (CS) 1000 mg/day	61 (91)
Para-aminosalicylic acid (PAS) 22gm/day	55 (82)
Clofazimine (CFZ) 300 mg/day	7 (10)
Amoxicillin-clavulanic Acid (AMX-CLV) 1500 mg/day	24 (36)
Clarithromycin (CLR) 1000 mg/day	3 (4)
**Antiretrovirals**	
Stavudine (D4T) 60 mg/day	23 (34)
Zidovudine (AZT) 600 mg/day	23 (34)
Lamivudine (3TC) 300 mg/day	59 (88)
Tenofovir (TDF) 300 mg/day	15 (22)
Abacavir (ABC) 600 mg/day	2 (3)
Nevirapine (NVP) 400 mg/day	15 (22)
Efavirenz (EFV) 600 mg/day	34 (51)
Lopinavir/ ritonavir (LPV/r) 800/200 mg/day	7 (10)
Atazanavir/ ritonavir (ATZ/r) 300/100 mg/day	2 (3)
Indinavir/ ritonavir (IND/r) 1600/200 mg/day	1(1)

* for at least two weeks.

The median CD4-count of the patients at the time of MDR-TB treatment initiation was 152 cells/μl (IQR: 91-220). Forty-four patients (65.7%) were on ART before a diagnosis of MDR-TB was made. Of the 23 subjects not on ART at the initiation of TB therapy, 19 were started a median of 1.1 months after MDR-TB treatment initiation (IQR: 1.1–4.7). Four patients never started on ART: one of them died and three were lost-to-follow-up. Fifty-three and ten patients were treated with first line, NNRTI-based ART and second-line protease-inhibitor-based ART respectively. Table 2 lists the ARV medication and dosages commonly used to treat HIV/MDR-TB patients in this program.

### Treatment outcomes

Treatment outcomes in this Mumbai cohort, including immunological response, expressed in CD4 gain, have been reported elsewhere [4]. Overall, by the end of 2011, among the 67 patients on treatment, 13 (19.4%) were successfully treated (i.e. cured or completed treatment), 14 patients (20.9%) died, nine defaulted (13.4%), two patients (3.0%) failed treatment and 29 patients (43.3%) were alive and on treatment.

### Adverse Events

Overall, 71%, 63% and 40% of patients experienced one or more mild, moderate or severe AE respectively. AE were most commonly attributed to cycloserine, ethionamide and *p*-aminosalicylic acid. There were 151 episodes of AE during the study period, 29 of them severe (Table 3). For patients who had more than one episodes of the same AE (e.g two or three episodes of gastrointestinal symptoms) we counted only one episode, the most severe of them. Among those who ever experienced an AE, the median number was two (range 1–6).

**Table 3:** Adverse events (episodes) and time of occurrence.

Adverse Event	Mild/n (%)*	Moderate n (%)*	Severen (%)*	Totaln (%)*	Time (weeks) median (IQR)
**Gastrointestinal symptoms**	16 (24)	8 (12)	6 (9)	**30 (45)**	**9 (3–19)**
**Peripheral neuropathy**	3 (4)	14 (21)	8 (12)	**25 (37)**	**16 (8–50)**
**Hypothyroidism/ Altered thyroid function tests**	21 (31)	0 (0)	0 (0)	**21 (31)**	**16 (12–29)**
**Psychiatric**	6 (9)	5 (7)	8 (12)	**19 (28)**	**8 (4–16)**
**Hypokalemia**	6 (9)	8 (12)	1 (1)	**15 (22)**	**16 (6–23)**
**Renal impairment**	6 (9)	6 (9)	2 (3)	**14 (21)**	**16 (8–24)**
**Loss of hearing**	1 (1)	3 (4)	3 (4)	**7 (10)**	**20 (8–20)**
**Seizures** (not graded)	-	-	-	**6 (9)**	**2 (1.5–4)**
**Arthralgia**	0 (0)	6 (9)	0 (0)	**6 (9)**	**13 (7–20)**
**Vestibular toxicity**	2 (3)	1 (1)	1 (1)	**4 (6)**	**6.5 (2.75–14**
**Tendinitis** (not graded)	_	_	_	**4 (6)**	**46 (25–61)**
**Hepatitis**	0 (0)	0 (0)	0 (0)	**0 (0)**	**N/A**

* (% of patients).

Life-threatening events were rare in this Mumbai cohort: only one patient experienced severe hypokalaemia eight weeks after treatment initiation and two patients (3.0%) experienced severe renal impairment, which was diagnosed after 16 and 24 weeks of therapy respectively. There were no instances of drug-induced hepatitis in this cohort.

Gastrointestinal symptoms were the most common AE; they occurred in nearly half of the patients, after a median (IQR) of nine weeks (3–19). Peripheral neuropathy occurred in 25 patients (37%); in eight, neuropathy was severe. Hypothyroidism was common, with nearly one third of the patients (31%) having altered thyroid function tests, at a median time of 16 weeks on DR-TB treatment. Psychiatric AE were also common and occurred early in treatment; almost one third of patients experienced psychiatric disturbances and in eight patients Cycloserine and/or Efavirenz had to be removed from the treatment regimen. Hypokalemia occurred commonly (22%) after a median of 16 weeks of treatment. Most AE occurred early, between the 2^nd^ and 4^th^ month of anti-TB treatment (Table 3).

All AE were initially managed symptomatically. Every effort was made to delay permanent discontinuation of any agent unless the AE was life-threatening or severe enough to interfere with treatment in spite of optimal management. The suspected offending drugs were reduced in dose or temporarily suspended. In fact, temporary suspension is what was done most frequently for a life-threatening event. Re-introduction was always attempted once symptoms, signs and/or laboratory results improved. Because the TB resistance profiles in this Mumbai cohort tended to be complicated with patients already having received most of the second-line anti-TB agents, finding an alternative agent to replace an offending drug was particularly challenging. Often, there were no alternative agents. Twenty-seven patients (40%) required permanent discontinuation of at least one offending drug (most frequently PAS and cycloserine) due to an adverse event. However, none of the patients had adverse reactions that led to indefinite suspension of the injectable agent or the entire MDR-TB treatment or antiretroviral therapy.

Eleven patients were hospitalized for AE during the study period. The main reasons for hospitalization were life-threatening events (severe renal impairment, hypokalaemia), seizures or severe psychiatric symptoms. Three patients died during hospitalization, eight patients were discharged recovered. Hospitalization was generally short (less than a week) and only two of the eleven patients had to be hospitalized more than once; both of them for hypokalaemia. Looking at final treatment outcomes, AE might have contributed to defaulting of two patients. For four patients, AE may have contributed to their deaths (two died with acute renal failure, one patient committed suicide and for one the cause of death is unknown), although we were not able to confirm this because the patients were severely ill and had other co-morbidities (namely: chronic renal disease, chronic pancreatitis, alcoholism, clinical depression and acute erythroderma).

The occurrence of AE did not differ significantly between men and women or between patients aged < = 36 years and older patients (p = 0.45 and 0.80, respectively). Similarly, no statistically significant difference was found between patients with pulmonary and extrapulmonary TB, patients who had previously received 2^nd^ line anti-tuberculosis agents or not, or between patients on first and second line ART.

In this cohort of HIV-infected patients on ART, toxicity due to antiretrovirals alone was uncommon. Discontinuation of complete antiretroviral treatment was never required. Of particular concern was the co-administration of tenofovir with aminoglycosides and capreomycin, and their associated risk of additive renal toxicity. Similarly, the co-administration of efavirenz and cycloserine potentially increases the risk of psychiatric AE. Finally, of concern was the co-administration of stavudine and ethionamide, cycloserine or high-dose isoniazid and their associated risk of peripheral neuropathy. However, only five of 34 patients on efavirenz developed severe psychiatric symptoms that required discontinuation of the drug. Among 15 patients on tenofovir and injectable anti-tuberculosis drugs, two had developed renal impairment that required removal of tenofovir from the antiretroviral regimen. Finally, for at least two patients with severe peripheral neuropathy it was considered that stavudine was likely responsible and was discontinued.

On univariate analysis, the occurrence of a severe AE was not significantly associated with unfavorable outcome [Odds Ratio: 1.12 (95% CI: 0.41–3.07)]. No significant associations were found between demographic and clinical characteristics and the occurrence of AE in univariate and multivariate analyses.

## Discussion

This is, to our knowledge, the first report on MDR-TB treatment AE from a cohort of HIV-infected patients on antiretroviral treatment in Asia. Despite India's large burden of MDR-TB and large absolute numbers of people living with HIV, there are discouragingly few reports of successful treatment programs from the sub-continent [4], [16], [17].

In this Mumbai cohort, adverse reactions to 2^nd^ line anti-TB treatment occurred frequently but were managed mostly on an out-patient basis. Although there have been concerns that complicated adverse reactions and additive toxicities would prove difficult to manage among MDR-TB/HIV co-infected patients on ART and second-line anti-TB treatment, our experience was rather encouraging. Treatment interruptions due to AE were indeed common; however, no co-infected patient in this cohort required permanent discontinuation of anti-TB treatment or ART. This is similar to the experience in programs having MDR-TB patients not co-infected with HIV, where toxicity-related complete discontinuation of treatment was rare [7], [10], [18], [19].

There are several findings of interest in this study. Severe AE in this Mumbai cohort of HIV-infected patients were relatively less common than that reported from a program in a high HIV prevalence setting in Southern Africa [3] but relatively more common than that reported from an HIV-infected cohort in Peru [12]. Seung et al reported that 92% (70/76) of patients in a Lesotho cohort experienced at least one serious AE or clinical complication during a follow-up period of one year. HIV-positive patients are known to have an increased incidence of AE to first-line TB drugs but it is not well established that the incidence of AE to second-line TB drugs may be increased among these patients as well [20], [21]. Specific AE, such as hypothyroidism, hypokalaemia, loss of hearing, seizures, tendonitis and arthralgia, were most likely due to second-line TB drugs. Others, such as peripheral neuropathy, psychosis, gastrointestinal symptoms and renal toxicity are known AE of second-line TB drugs, but can also be caused or exacerbated by other conditions (e.g. HIV-related peripheral neuropathy, co-morbidities like diabetes mellitus) or other drugs (e.g., stavudine, efavirenz, zidovudine, protease inhibitors, tenofovir) commonly administered to HIV-infected patients [5], [13], [14].

Some AE occurred surprisingly less frequently than might have been expected, based on prior experience. Of note, no hepatitis occurred in this cohort, while in a Peruvian co-infected cohort, it was relatively common (17.3%) [12]. Similar rates of psychiatric events but higher rates of gastrointestinal symptoms, peripheral neuropathy and hypothyroidism were recorded in this Mumbai cohort compared to the Peruvian cohort. Severe renal impairment, loss of hearing and severe peripheral neuropathy occurred much less frequently in this Indian cohort than in the Lesotho cohort: 3% vs. 21%, 4% vs. 36%, and 12% vs. 51% respectively. The lower than expected frequency of certain AE and discontinuation of therapy warrants further investigation. The differences between settings may reflect differences in drug regimens, their doses, pharmacokinetics or simply definitions of AE and their grades. We urgently need data from programmatic settings and standardized data collection to better understand AE patterns and risks occurring among co-infected patients on first and second line ART and second line anti-TB treatment.

Altered thyroid function tests and hypothyroidism were particularly frequent among these patients. We hypothesize that this finding partly reflected the systematic thyroid function monitoring in our setting. In most other programmatic settings, like Peru and Lesotho, monitoring of thyroid function was limited to symptomatic or high risk patients, and this AE might have been underreported [6], [8]. In fact, a very recent study from Lesotho revealed a very high rate of hypothyroidism among patients on MDR-TB treatment (69%) at 13 weeks of treatment, which highlights the fact that this AE may be more common than previously recognized [22]. In the Mumbai cohort, PAS and ethionamide were both frequently in the same treatment regimen and this may also explain the high prevalence of hypothyroidism.

Most of these Mumbai patients were living in extremely difficult socio-economic conditions; the large majority of them were slum dwellers. The diagnosis of co-infection was shocking to most patients and many of them were in a critical condition and had a poor prognosis at the time of treatment initiation. However, only two patients were diagnosed with depression at the outset of therapy and relatively few patients developed major depression during treatment Although counselling was provided to all patients, lack of standardized tools may have limited its effectiveness in supporting adherence.

In this cohort, many patients referred to the MSF clinic from the private health sector had already been on various TB treatment regimens for long periods and had often experienced treatment AE, not always appropriately managed. Overall, the management of tuberculosis in the private sector in India has reportedly been chaotic and unregulated [23], [24]. The risk for established toxicities among these patients may have been already high at the time of their enrolment in the program.

There are several limitations to this study. First, even though the study was done prospectively, the data were collected from a small cohort in a programmatic setting in a resource-constrained country. Our findings may not be applicable to other populations with HIV/MDR-TB co-infection. Second, even though Mumbai is a large, modern metropolis, access to affordable, quality diagnostic facilities was rather limited. Despite our efforts to provide systematic monitoring for most AE, we were not able to capture all of them, especially during the early years of the program. This may have led to an underestimation of the prevalence of some of the AE in this cohort, e.g. otoxicity, due to limited access to audiometry [11], [25], [26]. However, for other AE we were able to provide more intensive monitoring than was previously reported in the literature, e.g. thyroid function. Third, we acknowledge the lack of standardized definitions and a generally accepted grading system of AE. Under-reporting or over-reporting as well as reporting bias may have occurred, especially for events not defined by laboratory criteria. This may have prevented us, and other researchers, from accurately measuring the occurrence of adverse events and from allowing for reliable comparisons between patient populations and settings. An additional reason for under-reporting or underestimating AE may be due to the fact that many patients are still on treatment and therefore at risk for new AE. In spite of these limitations our data yield important information regarding the AE observed using second-line anti-tuberculosis agents and first and second line antiretrovirals, to treat MDR-TB and HIV in a resource-poor setting.

We believe that our findings are encouraging and provide some of the much needed evidence that co-administration of ART and second-line TB treatment rarely causes life-threatening AE or leads to permanent discontinuation of treatment. We hope these findings encourage wider treatment of co-infected patients since the global cohort of HIV/MDR-TB patients being treated is still discouragingly small.

We strongly advocate for clinical and operational research related to treatment of people co-infected with DR-TB and HIV and we are eagerly awaiting new, effective, safe, well tolerated and affordable drugs that would dramatically shorten the anti-TB treatment duration and that would be easily and safely co-administered with antiretrovirals, with the lowest pill-burden possible.

Meanwhile, concerns about severe AE in the management of HIV/MDR-TB co-infected patients with the current recommended regimens are justified; however, they should not cause delays in the urgently needed rapid scale-up of antiretroviral therapy and second line anti TB treatment.
